# Energy-aware Scheduling of Surveillance in Wireless Multimedia Sensor Networks

**DOI:** 10.3390/s100403100

**Published:** 2010-03-31

**Authors:** Xue Wang, Sheng Wang, Junjie Ma, Xinyao Sun

**Affiliations:** State Key Laboratory of Precision Measurement Technology and Instrument, Department of Precision Instruments, Tsinghua University, Beijing 100084, China; E-Mails: wang_sheng00@mails.tsinghua.edu.cn (S.W.); mjj@mails.tsinghua.edu.cn (J.J.M.); sxy05@mails.tsinghua.edu.cn (X.Y.S.)

**Keywords:** wireless multimedia sensor networks, energy-aware scheduling, target tracking

## Abstract

Wireless sensor networks involve a large number of sensor nodes with limited energy supply, which impacts the behavior of their application. In wireless multimedia sensor networks, sensor nodes are equipped with audio and visual information collection modules. Multimedia contents are ubiquitously retrieved in surveillance applications. To solve the energy problems during target surveillance with wireless multimedia sensor networks, an energy-aware sensor scheduling method is proposed in this paper. Sensor nodes which acquire acoustic signals are deployed randomly in the sensing fields. Target localization is based on the signal energy feature provided by multiple sensor nodes, employing particle swarm optimization (PSO). During the target surveillance procedure, sensor nodes are adaptively grouped in a totally distributed manner. Specially, the target motion information is extracted by a forecasting algorithm, which is based on the hidden Markov model (HMM). The forecasting results are utilized to awaken sensor node in the vicinity of future target position. According to the two properties, signal energy feature and residual energy, the sensor nodes decide whether to participate in target detection separately with a fuzzy control approach. Meanwhile, the local routing scheme of data transmission towards the observer is discussed. Experimental results demonstrate the efficiency of energy-aware scheduling of surveillance in wireless multimedia sensor network, where significant energy saving is achieved by the sensor awakening approach and data transmission paths are calculated with low computational complexity.

## 1. Introduction

Wireless sensor networks (WSNs) utilize a large number of intelligent sensor nodes to accomplish complicated surveillance tasks, such as target tracking. Sensor nodes have capability of sensing, processing and wireless communication. Recently, the availability of low-cost hardware such as CMOS cameras and microphones has fostered the development of wireless multimedia sensor networks (WMSNs) [1]. Sensor nodes can ubiquitously retrieve multimedia content such as video and audio streams from the environment. The application of WMSNs is an attractive research area, however, some challenges exist. One important challenge is the energy problem. As sensor nodes are integrated into the unsupervised sensing field, the recharge of energy supplies is not feasible. Still, WMSNs should implement long-term applications. Therefore, the energy usage of sensor nodes should be well managed. As a typical application for WMSN, target surveillance is mainly studied here. Sensor nodes utilize microphone sensors to acquire acoustic signals, the energy feature of which provides the hint of target position. Importantly, a totally distributed network structure is discussed, that is, sensor nodes are randomly deployed and adaptively organized without any centralized controller, such as sink node. That is because the centralized controller which is usually fixed and faces the threat of attacks might become the bottleneck for the scalability and security of WMSN [2]. This distributed network structure will provide the resilience and robustness for the WMSN application and also reduce the high overheads of management. Meanwhile, the sleep mode of sensor nodes is considered, where the components of sensor nodes can be shut down when there is no sensing, processing and communication task. As described in [3], the power consumption of sleep mode is usually several orders of magnitude less than that of active mode. Sensor nodes can be sent to sleep and awakened by messages, switching between active and sleep modes. Then, the approaches for target surveillance will be investigated.

To satisfy the target detection accuracy, the sensor nodes around the target should be grouped for collaborative sensing. Considering a sensor scheduling approach, sensor nodes go to sleep for energy saving and wake up for potential target detection. Thereby, the prior target motion information can be adopted to schedule the mode of sensor nodes. Considering the maneuvers of the target, its trajectory should be tracked in case of a missing event. As the state model of target motion is nonlinear, target tracking is usually treated as a nonlinear estimation problem. There are several classical approaches, such as Extended Kalman filter (KF) [4] and particle filter (PF) [5]. However, the computational complexity hinders their wide application in WMSN. Actually, it was proved in [6] that the problem of maneuvering target tracking can be viewed as a problem of adaptive time series prediction. Then the approach of hidden Markov model (HMM) can also be introduced to solve the tracking problem, since HMM is a widely-used tool to analyze and forecast time series phenomena. HMM has been used successfully to analyze various types of time series including DNA sequence analysis and speech signal recognition [7]. Some existing literatures in fact adopted HMM to predict the state of moving targets [8,9]. During target tracking, the stochastic varying of target acceleration should be tracked. Compared to other forecasting method, such as Radial basis function network (RBFN) [10], HMM can approximate the stochastic process easily.

With the prior target motion information, sensor nodes can be adaptively grouped to accomplish target localization. There are two approaches for target localization with acoustic signals received by sensor nodes: estimate of time delay of arrival (TDOA) and estimate of energy attenuation [11]. The latter is preferred here for its low computational complexity. Concerning the energy consumption of target localization, a subset of sensor node around the target is selected to perform the target detection. Sensor nodes may decide whether to participate in target detection separately so that the management overheads are reduced. Also, the energy capacity of each sensor node should be taken into account. For acoustic signal from selected sensor nodes, target localization can be treated as a two dimensional search problem. Particle swarm optimization (PSO) is an efficient tool for solving combinatorial optimization and dynamic optimization problems in multi-dimensional space, which implements fast convergence and good robustness [12]. PSO is considered to search for the target position.

Moreover, an observer which may be located at any position in the sensing field will query for the information about the target trajectory. Under the distributed network structure, a routing scheme for data transmission to the observer should be exploited. Lowest cost path, which has the lowest transmission overheads, is considered here. To simplify the routing algorithm, the sensor nodes which are not likely to take part in the lowest cost path should be eliminated from the path search procedure.

Focusing on the above-mentioned problems in target surveillance applications of WMSN, this paper proposes an energy-aware sensor scheduling method. A totally distributed network structure is adopted. An energy attenuation model is presented to model the propagation characteristics of acoustic signals. Then, an energy consumption model is introduced to describe the active and sleep modes of sensor nodes. The energy-aware sensor scheduling involves three aspects: target forecasting, target localization and data report. Target forecasting is performed by a HMM-base algorithm, where HMM approximates the stochastic varying factor of target acceleration. With the future target position, sensor nodes around the target can be awakened for target detection and localization. Here, a fuzzy control approach is utilized to select a group of sensor nodes according to the expected signal energy feature and residual energy of sensor nodes. Thus, target localization can be accomplished by PSO with signal energy feature provided by these sensor nodes. Besides, a local routing scheme with low computational complexity is exploited for data transmission towards the observer. Experiment analysis is presented to demonstrate the efficiency of the proposed energy-aware scheduling method, where target localization accuracy and energy saving of WMSN are evaluated.

The rest of this paper is organized as follows. Section 2 gives an overview of the related work. Section 3 describes the distributed network structure and the basic models, including energy attenuation model of acoustic signal and energy consumption model of sensor node. In Section 4, the energy-aware sensor scheduling method will be presented, where the HMM-based target forecasting, energy-aware target localization and local data report routing are introduced respectively. The experiments results are presented in Section 5, where the efficiency of energy-aware sensor scheduling method is evaluated in target surveillance application of WMSN. We conclude this paper in Section 6.

## 2. Related Work

Recently, WSN has become an attractive research area for its wide civil and military applications. Still, there are several bottlenecks for its practical application. One of the most critical problems is the energy constraint of sensor nodes. Therefore, some optimization methods for reasonable energy usage are exploited, referring to all the layers of WSN architecture [13].

Since the WSN is application-oriented, we choose target surveillance for investigation. Some energy-aware approaches have been proposed for target surveillance application. In [14], a tree-based algorithm is proposed to facilitate collaborative tracking of moving targets. The authors of [15] and [16] both make efforts on the particle filtering style target tracking algorithm using binary sensors, which can detect whether an object is approaching or not. Besides, a distributed tracking system based on extended Kalman filter techniques is implemented in [17]. Based on an information-driven approach, a tracking system is built in [18] with distributed Bayesian estimation, given previous estimation and new sensor inputs. In the network structure to be discussed, there is no super node with high processing power. Thus, these tracking algorithms may be computational-expensive for sensor nodes.

In WMSN, sensor nodes can capture and process multimedia data, such as video and audio streams. There are some researches on target surveillance application of WMSN. In [19], an environmental monitoring system is provided to record animal behaviors for a long period of time. The shooter localization system collects the time stamps of the acoustic detection from different nodes within the network to localize the positions of the snipers [20]. The Line-in-the-Sand project focuses on target tracking and classification [21]. Here, microphone sensors are adopted to localize the target. Localization problems with acoustic signal are overviewed in [22]. We adopt the energy attenuation model of acoustic signal for target localization, because it is relatively easy to establish and less influenced by environmental changes.

More importantly, the energy management of sensor node is usually of concern, because the modules of a sensor node can be well controlled by their operating system now. An energy management protocol for target tracking is proposed in [23], where the sensor nodes far away from the target are allowed to sleep. However, there is not enough effort made on extracting target motion information and the target detection performance with multiple sensor nodes has not been discussed.

In addition, lowest cost paths have been utilized in data collection and dispatch. Dijkstra’s algorithm is usually employed [24,25]. However, its global path search for each sensor node will lead to high computational cost. Therefore, an approach should be investigated to reduce the number sensor node included in the path search procedure.

The difference of our work mainly involves: (a) sensor nodes are randomly deployed and adaptively organized without any centralized controller; (b) a HMM-based target forecasting approach is proposed; (c) sensor nodes are scheduled according to the prior target motion information for energy saving; (d) energy-aware sensor node selection is performed with fuzzy control approach; (e) target localization is implemented by PSO; (f) a local routing scheme with low computational complexity is exploited for data transmission towards an observer.

## 3. Preliminaries

It is assumed that the WMSN is composed of stationary sensor nodes, which are deployed randomly in a two dimensional sensing field. Sensor nodes utilize microphone sensors to capture acoustic signals from the potential vehicle target. The functionality of microphone sensor as well as the attenuation of acoustic signal will be described. Besides, the active and sleep modes of sensor node will also be defined.

### 3.1. Energy Attenuation Model of Acoustic Signal

Each sensor node is equipped with one microphone. For the vehicle target, the sound of engine can be acquired by the sensor nodes. For these passive sensors, the sensing range is not only determined by their sensitivity, but also strongly influenced by the intensity of acoustic signal produced by the target. In [26], it has shown that the effective sensing range of microphones in vehicle detection is nearly 100 m. Here, the sensing range of sensor node is assumed as 60 m.

The way that acoustic signal propagates with the distance from the source is dependent on the size and shape of the source, the surrounding environment, prevailing air currents and the frequencies of the propagating acoustic signal [27]. To simplify the problem, it is assumed that the acoustic signal propagation in the free air and the acoustic source is treated as an omni-directional point.

The acoustic signal propagation is discussed in the two dimensional sensing field. Suppose that the source position of acoustic signal (target position) is denoted by *p_s_* = [*x_s_*,*y_s_*]*^T^*. The acoustic signal at *p_s_* is denoted by *u_s_* (*t*). Two dimensional propagation of the acoustic signal is mathematically represented by [22]. The acoustic signal at the position *p_d_* = [*x_d_*,*y_d_*]*^T^* can be calculated as:

(1) $${u^{\prime }}_{d}(t)=\frac{1}{4\pi \left\|p_{s}-p_{d}\right\|}u_{s}\left(t-t_{sd}\right)$$

where ‖ · ‖ represents the vector 2-norm, which is defined as:

(2) $$\left\|{\left[x,y\right]}^{T}\right\|=\sqrt{x^{2}+y^{2}}$$

*t_sd_* denotes the propagation time of acoustic signal from position *p_s_* to *p_d_*, which is calculated as:

(3) $$t_{sd}=\frac{\left\|p_{s}-p_{d}\right\|}{c}$$

where *c* is propagation velocity of acoustic signal. It can be taken as a constant in this case. *t_sd_* is an important parameter in the TDOA approach.

For the sensor node located at position *p_d_*, the acoustic signal is represented as:

(4) $$u_{d}(t)=\frac{\alpha_{d}}{4\pi \left\|p_{s}-p_{d}\right\|}u_{s}\left(t-t_{sd}\right)+n_{d}(t)$$

where the gain of microphone is denoted by *α_d_*. *n_d_* (*t*) is the zero-mean additive white Gaussian noise from the environment, which is assumed to be uncorrelated with the source acoustic signal.

Equation (4) is a comprehensive description of the propagating characteristics of acoustic signals. Information of the acoustic source can be inferred from this equation. Specifically the problem is to estimate *p_s_* from the acoustic signal *u_d_* (*t*) sensed by the sensor node. When an acoustic signal is propagating, it essentially propagating energy emitted from the source. For the acoustic sensed by the sensor node, the energy feature is denoted by $E\left[u_{d}^{2}(t)\right]$. Here, *E*[·] means the expected value:

(5) $$E\left[u^{2}(t)\right]=\frac{\sum_{t=1}^{M_{w}}u^{2}(t)}{M_{w}}$$

where *u*(*t*) is the time series for a given acoustic signal and *M_w_* is the number of the sample points used for averaging the energy.

According to Equation (4), we have:

(6) $$E\left[u_{d}^{2}(t)\right]=\frac{\alpha_{d}^{2}}{16\pi^{2}{\left\|p_{s}-p_{d}\right\|}^{2}}E\left[u_{s}^{2}(t)\right]+E\left[n_{d}^{2}(t)\right]$$

where $E\left[u_{s}^{2}(t)\right]$ and $E\left[n_{d}^{2}(t)\right]$ are the expectation of energy for the source acoustic signal and noise, respectively. The signal-to-noise ratio can be calculated as:

(7) $$R_{SN}=\frac{E\left[u_{d}^{2}(t)\right]}{E\left[n_{d}^{2}(t)\right]}-1$$

Since the target is a truck, the second term is much smaller than the first term in Equation (6) when the target locates in the sensing range of microphone sensor. Still, the signal-to-noise ratio is an important factor for the accuracy of target localization. This will be further discussed in the future section. Here, the term $E\left[n_{d}^{2}(t)\right]$ is ignored to simplify the problem. Then the acoustic energy attenuation model can be described as:

(8) $$\frac{E\left[u_{d}^{2}(t)\right]}{E\left[u_{s}^{2}(t)\right]}=\frac{\alpha_{d}^{2}}{16\pi^{2}{\left\|p_{s}-p_{d}\right\|}^{2}}$$

*u_s_* (*t*) and *p_s_* are unknown here. If the target locates in the sensing range of *N_s_* sensor nodes, we have:

(9) $$\{\begin{array}{c} E^{\left(1\right)}{\left\|p_{s}-p_{d}^{\left(1\right)}\right\|}^{2}=E_{s}\\ E^{\left(2\right)}{\left\|p_{s}-p_{d}^{\left(2\right)}\right\|}^{2}=E_{s}\\ \vdots \\ E^{\left(N_{s}\right)}{\left\|p_{s}-p_{d}^{\left(N_{s}\right)}\right\|}^{2}=E_{s} \end{array}$$

where *E*^(*i*)^ denotes the signal energy feature provided by sensor node *i* (*i* = 1,2,⋯,*N_s_*), $p_{d}^{\left(i\right)}$ denotes the position of sensor node *i* (*i* = 1,2,⋯,*N_s_*), and *E_s_* denotes the constant $\alpha_{d}^{2}\cdot E\left[u_{s}^{2}(t)\right]/\left(16\pi^{2}\right)$. Thereby, the target position can be estimated by solving (9).

### 3.2. Energy Consumption Model of Sensor Node

In WMSNs, each sensor node mainly consists of four components: microphone sensor, processor, receiver and transmitter. For a real operational system, the control of each module is programmable by employing the corresponding hardware drivers in the application layer.

Each module of a sensor node has two operation states: high-power state and low-power state. In general, each module accomplishes its task in the high-power state while it is shut down to save energy in the low-power state. The power consumption of modules in different operation states are given in Table 1. Actually, according to the datasheet of MicaZ [28] and CC1100 [29], the current consumption in radio initialization cannot be neglected. However, compared to the operation time of other modules, the radio initialization time is quite little (only 240 μs from sleep to RX or TX mode for CC1100 [29]). Thus, the power consumption in such a short period can be negligible. From Table 1, it can be found that the microphone sensor, processor and receiver modules have constant power consumption in both high-power and low-power states. In particular, the low-power state for receiver module keeps an acronym for low-power listening. In this state, the radio can listen to the media with low-power consumption, by which the other modules of sensor node can be awakened if necessary [30].

For the transmitter module, the power consumption of the low-power state is constant while the power consumption of the high-power state which is denoted by *P_Tx_* depends on the data transmission procedure. When sensor node *i* transmits data to sensor node *j*, the power received by sensor node *j* has the following relation with the power transmitted by sensor node *i* according to the Friis free space propagation model [31]:

(10) $$P_{j}=P_{i}\frac{G_{t}G_{r}\lambda^{2}}{{\left(4\pi \right)}^{2}d_{i,j}^{2}\beta }$$

where *G_t_* is the gain of transmitter, *G_r_* is the gain of receiver, *λ* is the radio wavelength, and *β* is the system loss factor. The physical distance *d_i,j_* between sensor node *i* and *j* is computed from the locations. With the receiving power threshold *P_th_*, the transmission power *P_Tx_* can be calculated as:

(11) $$P_{Tx}=\alpha_{l}d_{i,j}^{2}$$

where *α_l_* denotes the constant ${\left(4\pi \right)}^{2}R_{c}^{2}\beta P_{\mathrm{th}}/\left(G_{t}G_{r}\lambda^{2}\right)$.

**Figure 1.**
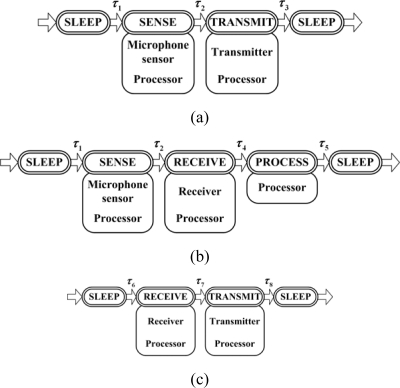
Three operation mode transition approach for sensor nodes: (a) Data acquisition node; (b) Data collection node; (c) Data forwarding node.

Then, operation modes of sensor nodes are defined according to the operation state of modules. There are five operation modes: SLEEP, SENSE, PROCESS, RECEIVE, and TRANSMIT. In the SLEEP mode, all the modules stay in the low-power state; the SENSE mode requires the microphone sensor and processor modules kept in the high-power state; the PROCESS mode requires the processor module staying in the high-power state; the RECEIVE mode requires the processor and receiver modules in the high-power state; the TRANSMIT mode requires the processor and transmitter modules both in the high-power state. Each sensor node may take part in the target surveillance application as a data acquisition node, a data collection node or a data forwarding node. Accordingly, three transition approaches are given as shown in Figure 1. A data acquisition node is awakened for sensing and transmitting the data. A data collection node will sense data and also receive data from other sensor nodes; after that, it accomplishes the data process task, such as target localization and forecasting. A data forwarding node is awakened to forward the data to the observer. In addition, there is latency when a sensor node transit from one operation mode to another, which is denoted by *τ_i_* (*i* = 1,2,⋯,8) in Figure 1. The value of latency is defined in Table 2. Here, the operation mode transition latency and power consumption are typical values referred to [32].

## 4. Energy-aware Sensor Scheduling Method

For the target surveillance purpose, a totally distributed WMSN will be discussed to provide flexible and adaptive capability. As stated in Section 3, the sensor nodes are randomly deployed. It would be a waste of energy to keep all the sensor nodes in active mode. Thus, an energy-aware sensor scheduling method is exploited here. It mainly involves three activities: target forecasting, target localization and data report. The future target position is obtained by target forecasting. Thus, the sensor nodes with potential sensing tasks can be awakened while the other sensor node can keep asleep to save energy. These sensor nodes are adaptively grouped with a fuzzy control approach. With the signal energy feature provided by sensor nodes, target localization is implemented with PSO algorithm. Besides, when any observer queries for the target trajectory information, it should be forwarded to the observer. A local routing scheme will be considered to reduce the computational cost.

### 4.1. HMM-based Target Forecasting

In WMSN, target localization is performed with constant sensing period *T*. For each sensing period, a data collection node which is randomly chosen from the awakened sensor nodes performs target forecasting after calculate the current target position. The historical target trajectory is delivered from one data collection node to another during the target surveillance procedure. The historical target trajectory and the current target position are utilized to estimate the prior target motion information. As the target localization task is time-critical and the computation resource of sensor node is limited, the forecasting algorithm should be light-weight. Here, a novel HMM-based algorithm is proposed.

HMM is built on the probabilistic framework for modeling a time series of multivariate observations [33]. It has strong statistical foundation, computational efficiency to develop and ability to predict similar patterns efficiently.

In the two dimensional field, the time series of historical target position is presented by Descartes coordinates. One direction of the target trajectory {*y_k_* | *k* = 1,2,⋯,*N_a_*} is taken for discussion. The problem is to estimate the target position *y*_*N*_*a*_+1_ at the next sensing period. The same forecasting approach can be implemented on the other direction. Here, the observation vector sequence is considered as follows:

(12) $$O_{k}={\left[O_{k}^{1},O_{k}^{2},O_{k}^{3},O_{k}^{4}\right]}^{T}={\left[\nabla^{3}y_{k},\nabla^{2}y_{k},\nabla y_{k},y_{k}\right]}^{T}$$

where ∇ is the differencing operator, which is defined as:

(13) $$\left\{ \begin{array}{l} \nabla y_{k}=y_{k}-y_{k-1}\\ \nabla^{2}y_{k}=\nabla y_{k}-\nabla y_{k-1}=y_{k}-2y_{k-1}+y_{k-2}\\ \nabla^{3}y_{k}=\nabla^{2}y_{k}-\nabla^{2}y_{k-1}=y_{k}-3y_{k-1}+3y_{k-2}-y_{k-3} \end{array}\right.$$

Thus, ∇*y*_*k*_ denotes the target velocity factor, ∇^2^*y*_*k*_ denotes the target acceleration factor, and ∇^2^*y*_*k*_ denotes the varying factor of target acceleration. The hidden state set is denoted by $S_{m}=\left\{s_{m}^{1},s_{m}^{2},\cdots ,s_{m}^{N_{m}}\right\}$, where *N_m_* is the number of hidden states.

Then a HMM can be constructed as:

(14) $$\lambda_{m}=\left\{A_{m},B_{m},\pi_{m}\right\}$$

where *A_m_* is the transition matrix of the hidden state, *B_m_* = {*b_m_*} is the observation emission matrix and *π_m_* is the prior probability matrix. The parameter estimation of HMM can be approached as a maximum likelihood estimation (MLE) problem.

The Viterbi algorithm [34] is adopted to estimate optimal hidden state sequence $Q_{m}=\left\{q_{m}^{1},q_{m}^{2},\cdots ,q_{m}^{N_{a}}\right\}$. According to the Markovian process, if $q_{m}^{N_{a}}=s_{m}^{i}$, then the forecasting can be performed as:

(15) $$Q_{N_{a}+1}=\sum_{j=1}^{N_{m}}A(i,j)E\left(b_{j}\right)$$

where the *b_i_* is probability distribution of observation vector for the hidden state $s_{m}^{i}$ and *E*(·) means the expected value. Then, $O_{k}^{4}$ is forecasting result for target position.

For comparison, another forecasting approach of RBFN is considered. RBFN is a three-layer feed-forward neural network which is embedded with several radial-basis functions. Such a network is characterized by an input layer, a single layer of nonlinear processing neurons, and an output layer. The output of the RBFN is calculated according to [35]:

(16) $$z_{\mathrm{out}}=\sum_{j=1}^{M}\omega_{j}\chi_{j}\left({\left\|z_{in}-c_{j}\right\|}_{2}\right)$$

where *z_in_* is an input vector, *χ_j_* is a basis function, ‖ · ‖_2_ denotes the Euclidean norm, *ω_j_* are the weights in the output layer, *M* is the number of neurons in the hidden layer, and *c_j_* are the centers of RBF in the input vector space. The functional form of *χ_j_* is assumed to have been given, which is always assumed as Gaussian function:

(17) $$\chi \left(z_{\mathrm{in}}\right)=e^{-\frac{z_{\mathrm{in}}^{2}}{\sigma^{2}}}$$

where *σ* is a constant.

Here, the target position is usually forecasted as:

(18) $$y_{N_{t}+1}=\sum_{j=1}^{M}\omega_{j}\chi_{j}\left({\left\|y_{N_{t}-2:N_{t}}-c_{j}\right\|}_{2}\right)$$

The performance of the two approaches will be compared with the experiments.

### 4.2. Energy-aware Target Localization

**Figure. 2.**
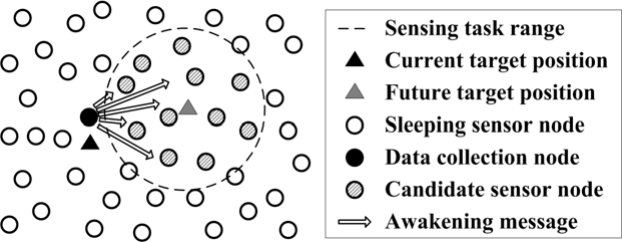
Sensor node awakening with the future target position.

Given the future target position, the sensor nodes with potential sensing tasks are known. As shown in Figure 2, the current data collection node multicasts an awakening message to the sensor nodes which are located in the sensing task range. The architecture is similar to the cross-layer architecture proposed in [36]. These sensor nodes are defined as candidates for target detection. Meanwhile, the other sensor nodes remain in sleep mode. The awakening message involves the time information. Thereby, the sensor nodes wake up for detection after the same time interval so that time synchronization can be easily achieved. Here, the communication energy consumption for awakening and time synchronization is ignored as the size of these messages is small.

**Figure 3.**
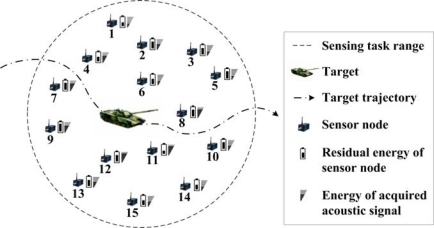
Two metrics of sensor nodes in the sensing task range.

As sensor nodes are usually deployed with high density, it is not necessary for all the sensor nodes to acquire the target information. Thus, the sensor nodes might be grouped in the sensing task range. As shown in Figure 3, two metrics of sensor nodes in the sensing range is concerned: residual energy of sensor node and the energy of acquired acoustic signal. The problem is to select a group of sensor nodes to implement the target localization [37]. In [37], a target direction-based sleep scheduling (TDSS) algorithm was proposed to achieve sensor nodes selection in the process of target tracking, and it was also proved that TDSS algorithm can minimize the energy consumption and guarantee the tracking accuracy. However, TDSS is a centralized algorithm, and it depends on a special assumption of the sensor node’s working mode. Obviously, centralized decision approach will lead heavy communication overheads. Thus, it is not applicable in resource-constrained WSN. To solve this problem, a fuzzy control approach is proposed to select the sensor nodes in a distributed way.

Fuzzy control is viewed as a branch of intelligent control which serves as an emulator of human decision-making behavior that is approximate rather than exact [38]. It will provide robustness for the sensor node selection. The fuzzy control approach includes three stages: receipt, judgment and action.

In the receipt stage, sensor nodes in the awakening range obtain global information from the awakening message. It is assumed that there are *N_c_* candidate sensor nodes in the sensing task range. The awakening message which the data collection node multicast should contains three values: the first one is the average residual energy *Ē^R^* of sensor nodes in WMSN; the second one is the normalized signal energy feature $E_{i}^{A}$ (*i* = 1,2,⋯,*N_c_*); the third one is the average signal energy feature *Ē^A^*. The average residual energy *Ē^R^* is delivered together with the historical target trajectory from one data collection node to another. As the data collection node has the knowledge of the detecting sensor node number, the average residual energy *Ē^R^* can be updated as:

(19) $${\bar{E}}^{R}={\bar{E}}^{R}-\frac{N_{s}{}^{\prime }}{N}\Delta E$$

where *N*′*_s_* is an estimation of required detecting sensor node number, *N* is the total sensor node number in WMSN, and Δ*E* is the energy consumption for one detecting sensor node in one sensing period. Here, *N*′*_s_* is a predefined parameter to denote that the target can be well localized with a certain number of sensor nodes. It is experientially determined by a large number of results of target localization. The total sensor node number is a constant recorded by each sensor node in the initialization of sensor network. And Δ*E* can be estimated according to the energy consumption model of sensor node. The normalized signal energy feature $E_{i}^{A}$ (*i* = 1,2,⋯,*N_c_*) is calculated by the data collection node according to the future target position and the energy attenuation model:

(20) $$E_{i}^{A}=\frac{\underset{i=1,2,\cdots ,N_{c}}{\text{min}}{\left\|p_{s}-p_{d}^{\left(i\right)}\right\|}^{2}}{{\left\|p_{s}-p_{d}^{\left(i\right)}\right\|}^{2}}$$

where *p_s_* is the future target position and $p_{d}^{\left(i\right)}$ is the position of candidate sensor node *i*. $E_{i}^{A}$ belongs to the interval (0,1]. Beside, the average signal energy feature is calculated as:

(21) $${\bar{E}}^{A}=\frac{\underset{i=1,2,\cdots ,N_{c}}{\text{min}}{\left\|p_{s}-p_{d}^{\left(i\right)}\right\|}^{2}}{0.25R_{s}{}^{2}}$$

where *R_s_* is the sensing range of microphone sensor.

**Figure 4.**
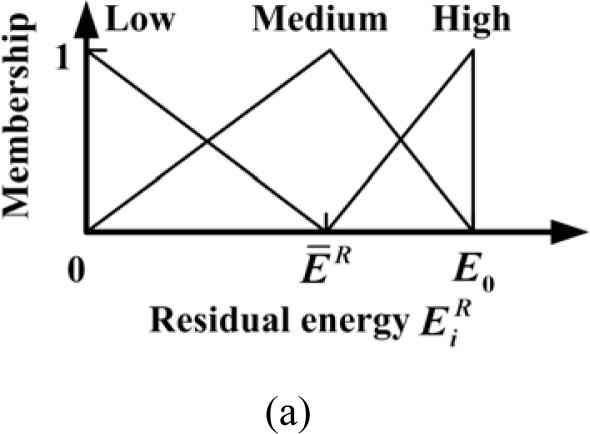

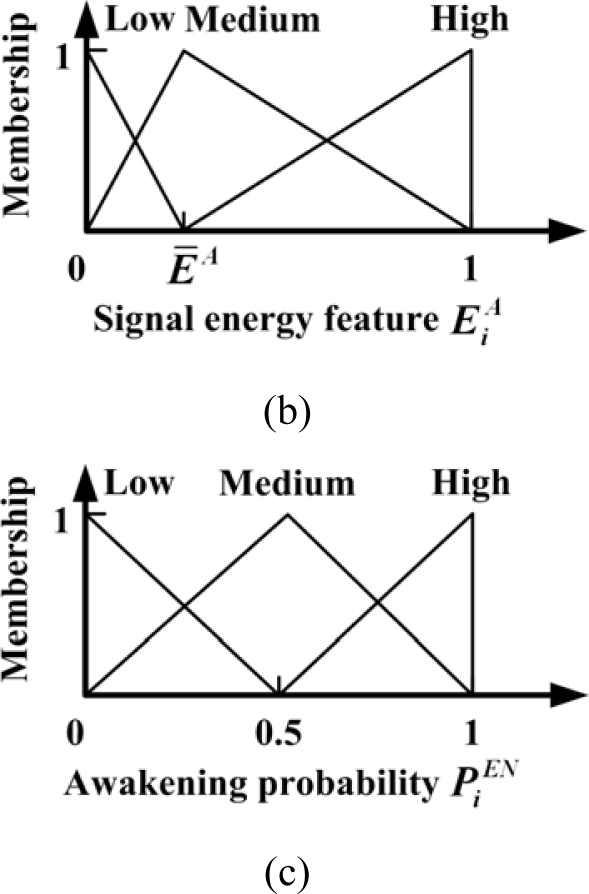
Fuzzy division of inputs and output for the fuzzy control approach: (a) residual energy; (b) signal energy feature; (c) awakening probability.

The fuzzy control system is constructed on each candidate sensor node in the judgment stage. The inputs of the fuzzy control system are the residual energy $E_{i}^{R}$ and the signal energy feature $E_{i}^{A}$, which are already known by the according candidate sensor node *i*. The output of the fuzzy control system is the awakening probability $P_{i}^{EN}$ of candidate sensor node *i*. The fuzzy division of the inputs and output is shown in Figure 4.

There are three linguistic variables (*low*, *medium* and *high*) for the residual energy $E_{i}^{R}$, the signal energy feature $E_{i}^{A}$ and the awakening probability $P_{i}^{\mathrm{EN}}$ respectively. The triangle membership functions are utilized here. Especially, the linguistic variable *medium* for residual energy $E_{i}^{R}$ and signal energy feature $E_{i}^{A}$ depends on the average residual energy *Ē^R^* and average signal energy feature *Ē^A^*, respectively. That is because they are relative values which vary during the target application of WMSN. Then the fuzzy rules should be designed. Energy and accuracy factors should be considered in the rules. For example, a sensor node with too little residual energy should remain in sleep mode although it is close to the target (such as sensor node 11 in Figure 3), and the sensor node which has medium residual energy and low signal energy feature should also keep in the sleep mode (such as sensor node 1 in Figure 3). Thus, the fuzzy rules are defined in Table 3. With the inputs and the fuzzy rules, each candidate sensor node can obtain an output of awakening probability $P_{i}^{\mathrm{EN}}$.

In the action stage of the fuzzy control approach, each candidate sensor node easily generates an random number *RAND_i_*. If $P_{i}^{\mathrm{EN}}<{\mathrm{RAND}}_{i}$, then it will participate in target detection at the next sensing instant; otherwise, it keep in the sleep mode.

In this way, the fuzzy control approach selects a group of sensor node adaptively, which will accomplish the target localization task. Moreover, one sensor node is chosen randomly from among them to act as the new data collection node. The old data collection node will deliver the historical target trajectory and average residual energy of the sensor nodes to the new one. Meanwhile, the other sensor nodes act as data acquisition node. Actually, the data collection node is used for collecting the data from the selected detecting sensor nodes and analyzing the position of target, then predicting the next position of target and sending out the awakening messages. All of the operations will consume overhead energy cost and network usage. Thus, if the data collection node is fixedly appointed, it will die out more quickly then other detecting sensor nodes. Random selection of data collection node can avoid this problem, and random selection will not bring in overhead cost. Compared to the purposeful selection, random selection of data collection node seems a little opportunistic without explicit performance guarantee. But the process of the selection of detecting sensor node can provide a guarantee for the lifetime of sensor network, because the data collection node is selected from the detecting sensor node. To achieve the tradeoff between the cost and the performance, random selection is preferred in this process. Furthermore, in the future work, a self-recommendation of data collection node will be investigated to improve this process.

It is assumed that there are *N_s_* detecting sensor nodes selected in the fuzzy control approach, which calculate the energy feature of acoustic signals according to (5) and send it to the data collection node. Thus, the data collection node keeps the signal energy feature $E_{i}^{\mathrm{AC}}$ and the corresponding sensor node positions $L_{i}^{d}$, where *i* = 1,2,⋯,*N_s_*. Then, target localization is carried out. In fact, there are many related works in the research field of target localization. However, depending on different motivations, different approaches carry out target localization with different sensing models, such as the center of gravity and DV-hop, and so on. In this paper, target localization is achieved by multiple acoustic sensor nodes. According to the analysis of the acoustic target localization in Section 3.1, this problem can be denoted by the over-determined equations in Equation (9). Although target localization can also be implemented by other approaches, the specific assumptions of signal models determine that PSO is an appropriate choice for our application. Moreover, compare to other solutions for over-determined equations, PSO is easy to implement and it is also a well known tool for combinatorial and dynamic optimization problems. By transforming the target localization problem into a two dimensional search problem, PSO can convincingly provide accurate localization results. Importantly, PSO is easy to implement in distributed computing paradigm, which implies that it is more suitable and scalable for resource-constrained WSN.

Kennedy *et al.* developed PSO based on the analogy of swarming of birds and fish schools in 1995. It is an efficient tool for solving combinatorial optimization and dynamic optimization problems in multidimensional space, which implements fast convergence and good robust.

The population of particles is set as *m*. For the particle *i*, *X_i_* = (*x*_*i*1_,*x*_*i*2_) represents its current position, where the elements present ordinates of potential target position and *V_i_* =(*v*_*i*1_, *v*_*i*2_) represents its current velocity; furthermore, *Pi* = (*p*_*i*1_,*p*_*i*2_) represents the best position it has achieved so far (*i* = 1,2,⋯*m*). As the optimization purpose is to solve (9), the minimization objective function *f*(*X*) is defined as follows:

(22) $$f(X)=\underset{i=1,2,\cdots ,N_{s}}{\text{var}}\left(E_{i}^{\mathrm{AC}}{\left\|X-L_{i}^{d}\right\|}^{2}\right)$$

where var is a function to compute variance.

The overall best position of the particle *i* can be calculated as follows:

(23) $$P_{i}(t+1)=\{\begin{array}{cc} P_{i}(t) & f\left[X_{i}(t+1)\right]\geq f\left[P_{i}(t)\right]\\ X_{i}(t+1) & f\left[X_{i}(t+1)\right]<f\left[P_{i}(t)\right] \end{array}$$

where *t* is the iteration index.

The overall best position of particle *i* is calculated as follows:

(24) $$P_{g}(t)=\text{min}\left\{f\left[P_{0}(t)\right],f\left[P_{1}(t)\right],\cdots ,f\left[P_{m}(t)\right]\right\}$$

Velocity and position of particle *i* are updated according to equations (25) and (26) respectively.

(25) $$v_{\mathrm{ij}}(t+1)=\omega (t)\times v_{\mathrm{ij}}(t)+c_{1}r_{1j}(t)\left[p_{\mathrm{ij}}(t)-x_{\mathrm{ij}}(t)\right]+c_{2}r_{2j}(t)\left[p_{\mathrm{gj}}(t)-x_{\mathrm{ij}}(t)\right]$$

(26) $$x_{\mathrm{ij}}\left(t+1\right)=x_{\mathrm{ij}}\left(t\right)+v_{\mathrm{ij}}\left(t+1\right)$$

where the index *j* = 1,2; *c*_1_ and *c*_2_ are acceleration constants, representing the weight of the stochastic acceleration terms that pull each particle toward the local best position and global best position; *r*_1*j*_ ∼ *U*(0,1) and *r*_2*j*_ ∼ *U*(0,1) are two separate random functions; *ω*(*t*) is the inertia weight, used to balance the global and local search ability. Here, a large inertia weight facilitates a global search while a small inertia weight facilitates a local search. As shown in (27), inertia weight linearly decreases through the course of the run. Accordingly, the optimization process can converge to the neighborhood of the global optimal solution smoothly at the prophase, and converge to the global optimal solution quickly at the anaphase.

(27) $$\omega (t)=0.9-\frac{t}{\mathrm{MaxNumber}}\times 0.5$$

where *MaxNumber* is the number of maximum iterations. After *MaxNumber* iterations, the optimal particle position presents the target localization result.

### 4.3. Local Data Report Routing Between Appointed Source Sensor Node and Destination Sensor Node

The purpose of target surveillance is to provide the target trajectory information for the observers. The observer might be located in any position of the sensing field. It injects a query message into the network through the closest sensor node, which is called destination sensor node. Then the destination sensor node will spread the query message simply by flooding. After the current data collection node receives the query message, it acquires the position of the destination sensor node. Here, the data collection node is called the source sensor node. The target trajectory data should be forwarded from the source sensor node to the destination sensor node and finally sent to the observer. Moreover, the data transmission between two data collection nodes and between the data collection node and detecting sensor node also needs the local data report routing. How to determine an efficient routing solution between the appointed source and destination sensor node is important for the cost of sensor network.

To obtain the optimal communication path, Dijkstra’s algorithm is usually considered [39]. However, the computation cost will grow fast when the sensor node number increases. Hence, improvements should be made in the search procedure and a local routing scheme is introduced here.

**Figure 5.**
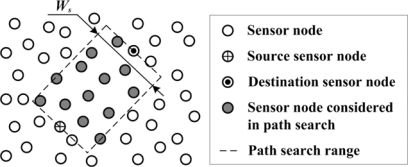
Local routing scheme for data report.

As shown in Figure 5, only the sensor nodes within the path search range are considered in the local routing scheme. The path search range is a rectangular area. Its length is equal to the distance between the source sensor node and the destination sensor node, while its width is denoted by *W_s_*. The direction of the long side of the rectangle is parallel to the orientation of a line segment from the source sensor node to the destination sensor node. The source sensor node and the destination sensor node are the midpoints of two rectangle short sides. Then, the path search is constrained in the local area. Here, *W_s_* should be well designed; this will be discussed in the experiment.

Then, the Dijkstra’s algorithm is performed on the path search area. The problem is formulated as:

- The destination sensor node is denoted by *p*_0_ and the set of sensor nodes within the path search range is denoted by *P* = {*p*_1_,*p*_2_,⋯,*p_n_*};
- According to equation (11), the edge weight between *p_i_* and *p_j_* is:
  (28) $$\omega_{\mathrm{ij}}=\frac{G_{t}\alpha_{l}d_{i,j}^{2}}{\alpha_{t}}+\Delta E_{c},\;i,j=0,1,2,\cdots ,n,\;i\neq j$$
  where *G_t_* is the data packet size, *α_t_* is the data rate, and Δ*E_c_* is the energy consumption for awakening a sensor node.
- Variable *D_i_* represents estimate of the lowest cost from *p_i_* to *p*_0_, and converges to the real value after iterations.
- The set of nodes that find the lowest cost paths is denoted by *Q*.

Searching procedure for the optimal data report path is described as follows:

- Initialize the network:
  (29) $$Q=\emptyset ,\;D_{0}=0,\;D_{i}=\omega_{i0},i=1,2,\cdots ,n$$
- Search for the next node with the lowest cost path to *p*_0_. For *p_i_ ∉ Q*, if *D_i_* satisfies:
  (30) $$D_{i}=\overset{n}{\underset{k=1}{\text{min}}}D_{k}$$
  The lowest cost path of *p_i_* is found, update *Q*:
  (31) $$Q=Q\cup \left\{p_{i}\right\}$$
  If *Q* = *P*, then search is completed. Oppositely, if *Q* ≠ *P*, continue searching.
- Update *D_j_* for all *p_j_* ∉ *Q* according to the result of step (ii):
  (32) $$D_{j}=\text{min}\left\{D_{j},\omega_{\mathrm{ji}}+D_{i}\right\}$$
  Continue to execute step (ii).

Iterate the step (ii) and (iii) until the lowest cost path from the source sensor node to the destination sensor node is determined. Hence, the data of target trajectory information is reported to the observer along the path which consists of several intermediate sensor nodes.

In conclusion, in the proposed algorithm, the process of target tracking, position prediction and new sensor nodes selection are implemented in turn. That is, the selected detecting sensor nodes and data collection sensor nodes are awaked after receiving the awakening message. Then they implement the signal acquisition and send the local results to data collection sensor nodes. After receiving the results of all detecting sensor nodes, the data collection sensor nodes carry out target localization by PSO algorithm. With the historical target trajectory and current estimated position, the future target position is predicted by HMM algorithm. Then the data collection sensor node sends out the awakening message to the sensor nodes which are located in the sensing task range. Then, a new circulation is started by selecting new detecting sensor nodes and data collection sensor nodes. From the above process, the working sensor nodes in each tracking step and sensing period can be determined according to the requirements of low energy consumption and high tracking accuracy.

## 5. Experimental Results

In this section, the efficiency of the proposed energy-aware sensor scheduling method will be evaluated in the target surveillance application of WMSN.

### 5.1. Experimental Environment

**Figure 6.**
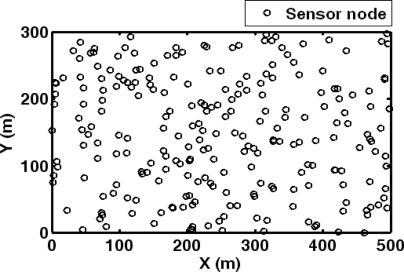
Sensor node deployment of WMSN.

It is assumed that sensor nodes are randomly deployed in a sensing field of 300 m × 500 m. To supply sufficient coverage of the sensing field, the sensor node is set as 240 after experimental analysis of communication range, sensing range of sensor node and the cost. Then, the sensor node deployment of WMSN is shown in Figure 6. The sensor nodes are unevenly deployed for investigating the performance of the proposed algorithm with different number of detecting sensor nodes in each time instant.

The sensing period for target detection is 0.5 s. In (10), the gain of transmitter *G_t_* = 2 the gain of receiver *G_r_* = 2, the radio wavelength *λ* = 0.125m, and the system loss factor *β* = 1. Besides, the receiving power threshold *P_th_* is defined as 6.04 × 10^−9^ W [40]. According to Figure 1, sensor nodes keep in the SENSE mode for 80 ms and keep in other modes for 10 ms respectively during operation mode transition.

The data rate of transmission is set as 400 kbit/s. The size of data packet involving the target trajectory information is 500 Byte. For each sensor node, the total energy consumption at time instant *t* can be calculated as:

(33) $$E_{\mathrm{total}}(t)=\int_{\tau =0}^{t}P(\tau )d\tau$$

where *P*(*τ*) means the power consumption at time instant *τ* for this sensor node. It must be noticed that the power consumption at each time instant includes the power consumption of each module of the sensor nodes, that is, microphone sensor, processor, receiver and transmitter. The total energy consumption is calculated by the predefined parameters in Table 1 and the above statement.

Thus, the residual energy can be calculated as:

(34) $$E_{r}(t)=E_{0}-E_{\mathrm{total}}(t)$$

where *E*_0_ denotes the initial energy of sensor node.

In the experiments, the data of acoustic signal is acquired by MICAz motes [28]. The MICAz is a 2.4 GHz, IEEE 802.15.4 compliant module used for WSNs. It is programmed to sample at the frequency of 4.96 kHz for acoustic signal acquisition. According the time of sensor node keeping in the SENSE mode, the size of signal data is 396. A truck is taken as the target.

### 5.2. Energy-aware Sensor Scheduling Experiment

**Figure 7.**
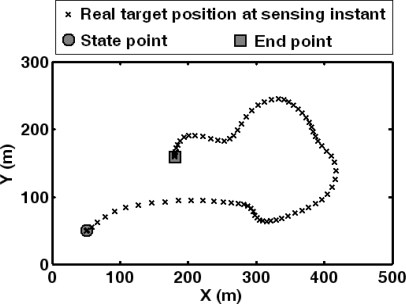
Target trajectory for forecasting in WMSN.

First, the performance of the HMM-based forecasting approach is analyzed. The target trajectory for forecasting is shown in Figure 7. The points on the trajectory are recorded according to the sensing period. The truck keeps moving in the sensing field for 40 s. Here, the moving trajectory of target is designed to encircle the sensing field of the sensor network, which guarantees that the experimental results are convincing for most network topologies with different densities.

**Figure 8.**
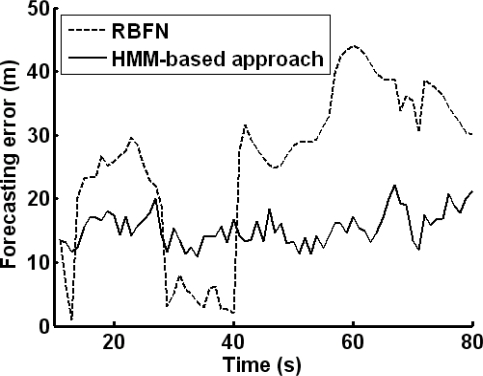
Forecasting error curves of the HMM-based approach and RBFN forecasting.

The HMM-based approach is compared with the RBFN forecasting. The forecasting error is shown in Figure 8. For the HMM-based approach, the forecasting error is smaller. That is because the stochastic component of target motion (especially, the varying factor of target acceleration) is estimated by HMM. The accuracy of target position forecasting will affect the performance of sensor node awakening and selection.

**Figure 9.**
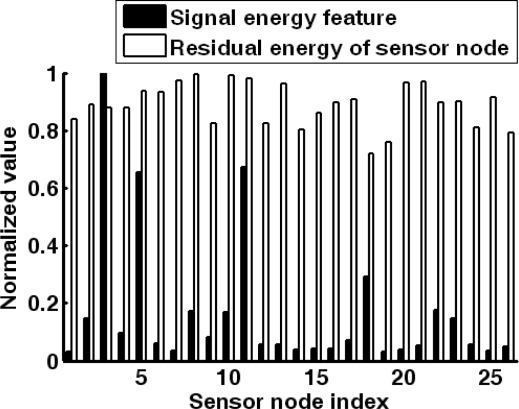
Normalized signal energy features and residual energy of the candidate sensor nodes when the forecasted target position is (173.54 m, 125.42 m).

With the forecasting result of target position, the efficiency of target localization will be evaluated. The target is designed to move randomly in the sensing field. In a specified sensing period, the forecasted target position is (173.54 m, 125.42 m). For the 26 candidate sensor nodes in the sensing task range, the normalized signal energy feature are given while the normalized residual energy is calculated according to the full energy capacity (as shown in Figure 9).

**Figure 10.**
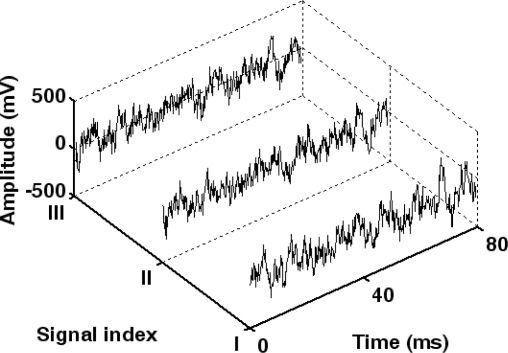
Acoustic signals acquired by sensor nodes 3, 5 and 11.

In this case, the average signal energy feature is 0.21 and the average residual energy of sensor node is 0.823. Utilizing the fuzzy control approach, seven sensor nodes are selected to accomplish the target detection. Their indexes are 3, 5, 8, 10, 11, 22 and 23. It can be found that the sensor nodes with too low signal energy feature or residual energy are eliminated, such as sensor node 6, 7 and 18. Thus, the efficiency of energy usage is enhanced. Then the seven sensor nodes are awakened to acquire the acoustic signals. The acoustic signals obtained by the three sensor nodes closest to the target are illustrated in Figure 10. Hence, the target position can be extracted from the energy feature of acoustic signals.

**Figure 11.**
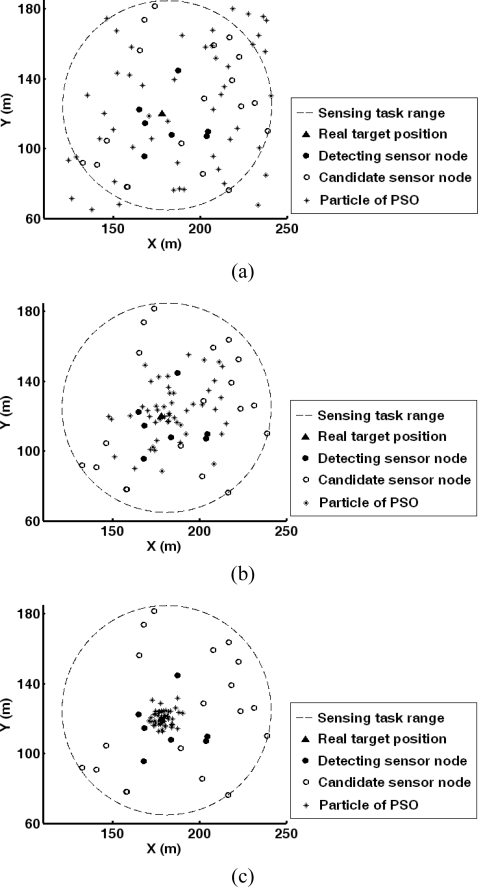
Two dimension search of particles during target localization with PSO approach: (a) Initial searching state; (b) Searching state after 5 iterations; (c) Searching state after 10 iterations.

**Figure 12.**
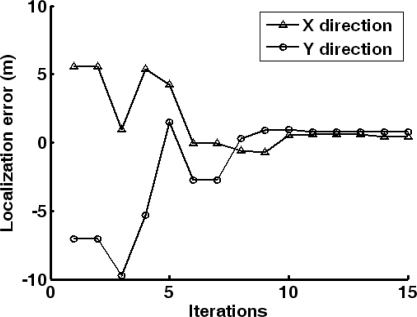
Convergence curves of localization error on x and y direction with PSO approach.

Adopting the energy feature of acoustic signals, the data collection node implements target localization. Here, PSO is utilized to search the target position. The search procedure is shown in Figure 11, where the initial particle positions as well as the swarm state after 5 and 10 iterations are given. It can be seen that the particle swarm converges to the real target position fast. Figure 12 shows the convergence curves of localization error on x and y direction during search procedure of PSO. It can be found that the algorithm converges to 10 within iteration.

**Figure 13.**
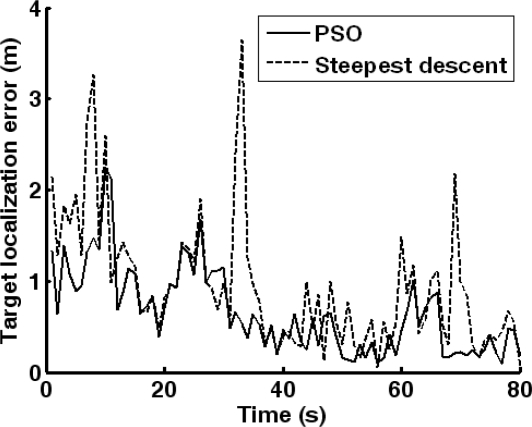
Localization performance comparison of PSO and steepest descent approaches.

With target trajectory of Figure 7, the performance of PSO and steepest descent approaches is compared. The curves of target localization error are shown in Figure 13. The PSO approach achieves higher accuracy than the steepest descent approach. As a heuristic algorithm, the PSO approach has better global search performance than greedy algorithms, such as steepest descent.

**Figure 14.**
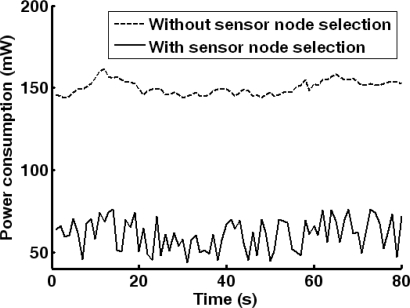
WMSN power consumption curves with and without sensor node selection.

Meanwhile, the energy consumption of WMSN is evaluated according to (33) and (34). The energy consumption with and without sensor node selection is compared in Figure 14. It can be found that significantly 59.4% energy is saved by sensor node selection.

**Figure 15.**
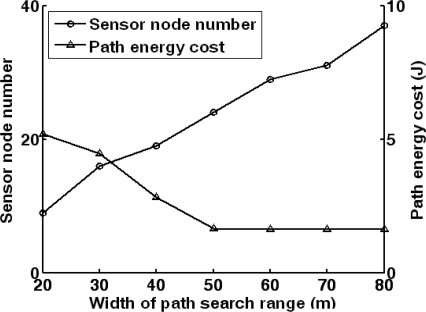
The number of sensor nodes considered in path searching and the energy cost of data report path adopting different width of path search range.

Finally, the local data report routing scheme is discussed. As mentioned in Section 4.3, its performance is influenced by the width of the path search range. As the sensor nodes are randomly deployed, the sensor nodes which are located at (47.61 m, 82.09 m) and (327.25 m, 137.03 m) are taken as the source sensor node and the destination sensor node respectively. Adjusting the width of path search range, the number of sensor nodes considered in path searching and the energy cost of data report path are illustrated in Figure 15. It can be found that it is easier to obtain the global optimal path when the width of data search range increases. Here, the width can be set as 50 m to achieve low-energy data report path with low computation cost. In the above experiments, the efficiency of energy-aware sensor scheduling is evaluated from three aspects: target forecasting, target localization and data report.

## 6. Conclusions

Focusing on the energy problem in the target surveillance application of WMSNs, this paper has proposed an energy-aware sensor scheduling method. In the method, a HMM-based approach is introduced to extract the prior target motion information. With the future target position, the sensor node are awakened and selected to detect the target in a distributed manner. Target localization with acoustic signals is investigated, where the fuzzy control approach and PSO algorithm are adopted. Moreover, a local data report routing scheme is utilized when there is any query from the observer. Experimental results verify that the proposed energy-aware sensor scheduling method achieves adequate localization accuracy and energy saving for WMSNs. For this paper, the main contribution is that a distributed network structure is employed for the WMSN, where sensor nodes are deployed randomly without any centralized control. Also, the approaches for target surveillance in WMSN are novel. The future research mainly includes: (a) implement the adaptive routing scheme on the sensor node of WMSN; (b) considering the mobility of sensor node in the target surveillance application; (c) investigate the statistical attributes of the proposed algorithm in the WSN with regular sensor nodes deployment.

## Figures and Tables

**Table 1.** Power consumption of modules in different operation states.

**Power consumption (mW)**	**High-power state**	**Low-power state**
Microphone sensor	1.73	0.003
Processor	24	0.03
Receiver	24	0.411
Transmitter	*P_Tx_*	0.1

**Table 2.** Operation mode transition latency for sensor node.

**Latency**	*τ*_1_	*τ*_2_	*τ*_3_	*τ*_4_	*τ*_5_	*τ*_6_	*τ*_7_	*τ*_8_
**Value (ms)**	1.2	3.2	2.7	2.5	0.2	2.7	0.2	2.7

**Table 3.** Fuzzy rules for sensor node selection.

**Awakening probability**$P_{i}^{\mathrm{EN}}$		**Residual energy**$E_{i}^{R}$		
		**Low**	**Medium**	**High**
Signal energy feature $E_{i}^{A}$	Low	Low	Low	Medium
	Medium	Low	Medium	High
	High	Medium	High	High

## References

[1] Akyildiz I.F., Melodia T., Chowdury K.R. (2007). A survey on wireless multimedia sensor networks. Comput. Netw.

[2] Liu K., Sayeed A.M. (2007). Type-based decentralized detection in wireless sensor networks. IEEE Trans. Signal Proc.

[3] Shin J., Chin M. Optimal transmission range for topology management in wireless sensor networks.

[4] Song C., Sharif H. Performance comparison of Kalman filter based approaches for energy efficiency in wireless sensor networks.

[5] Wang X., Wang S., Ma J. (2007). An improved particle filter for target tracking in sensor system. Sensors.

[6] Liu W., Farooq M. Tracking maneuvering targets *via* an ARMA type filter.

[7] Hassan M.R. (2007). A fusion model of HMM, ANN and GA for stock market forecasting. Expert Syst. Appl.

[8] Bruno M.G.S. (2004). Bayesian methods for multiaspect target tracking in image sequences. IEEE Trans. Signal Proc.

[9] Gai J., Li Y., Stevenson R.L. Coupled hidden Markov models for robust EO/IR target tracking.

[10] Lee M.J., Choi Y.K. (2004). An adaptive neurocontroller using RBFN for robot manipulators. IEEE Trans. Ind. Electron.

[11] Wang X., Bi D., Liang D., Wang S. (2007). Agent collaborative target localization and classification in wireless sensor networks. Sensors.

[12] Wang X., Wang S., Ma J. (2007). An improved co-evolutionary particle swarm optimization for wireless sensor networks with dynamic deployment. Sensors.

[13] Wang X., Ma J., Wang S., Bi D. (2007). Prediction-based dynamic energy management in wireless sensor networks. Sensors.

[14] Zhang W., Cao G. Optimizing tree reconfiguration for mobile target tracking in sensor networks.

[15] Aslam J., Butler Z., Crespi V. Tracking a moving object with a binary sensor network.

[16] Djuric P. M., Vemula M., Bugallo M.F. (2008). Target tracking by particle filtering in binary sensor networks. IEEE Trans. Signal Proc.

[17] Duh F.B., Lin C.T. (2004). Tracking a maneuvering target using neural fuzzy network. IEEE Trans. Syst. Man Cybern.

[18] Zhao F., Shin J., Reich J. (2002). Information-driven dynamic sensor collaboration for tracking applications. IEEE Signal Proc. Mag.

[19] Szewczyk R., Mainwaring A. An analysis of a large scale habit monitoring application.

[20] Simon G., Maroti M. Sensor network-based countersniper system.

[21] Arora A., Dutta P., Bapat S (2004). A line in the sand: a wireless sensor network for target detection, classification, and tracking. Comput. Netw.

[22] Li D., Hu Y.H. (2003). Energy based collaborative source localization using acoustic micro-sensor array. EURASIP J. Appl. Signal Proc.

[23] Du X., Lin F. Efficient energy management protocol for target tracking sensor networks.

[24] Noto M., Sato H. A method for the shortest path search by extended Dijkstra algorithm.

[25] Sudhakar T.D. Supply restoration in distribution networks using Dijkstra's algorithm.

[26] Durte M. F., Hu Y.H. (2004). Vehicle classification in distributed sensor networks. J. Parallel Distrib. Comput.

[27] Sheng X., Hu Y. Energy based acoustic source localization.

[28] (2006). MICAz Datasheet.

[29] (2009). CC1100 Datasheet.

[30] Wang X., Wang S. (2007). Collaborative signal processing for target tracking in distributed wireless sensor networks. J. Parallel Distrib. Comput.

[31] Wu Q., Rao N.S.V. (2004). On computing mobile agent routes for data fusion in distributed sensor networks. IEEE Trans. Knowl. Data Eng.

[32] Dutta P., Grimmer M., Arora A. Design of a wireless sensor network platform for detecting rare, random, and ephemeral events.

[33] González A.M., Roque A.M.S, Garcia-Gonzalez J. (2005). Modeling and forecasting electricity prices with input/output hidden Markov models. IEEE Trans. Power Syst.

[34] Rabiner L.R. (1989). A tutorial on hidden Markov models and selected applications in speech recognition. Proc. IEEE.

[35] Haykin S. (1999). Neural networks: a comprehensive foundation.

[36] Song L., Hatzinakos D. (2007). A cross-layer architecture of wireless sensor networks for target tracking. IEEE-ACM Trans. Netw.

[37] Jiang B., Han K., Ravindran B., Cho H. Energy efficient sleep scheduling based on moving directions in target tracking sensor networks.

[38] Lam H.K., Leung F. H. F. (2005). Stability analysis of fuzzy control systems subject to uncertain grades of membership. IEEE Trans. Syst. Man Cybern.

[39] Sobrinho J. L. (2002). Algebra and algorithms for QoS path computation and hop-by-hop routing in the Internet. IEEE-ACM Trans. Netw.

[40] Chen A., Lee D., Ch G. HIMAC: high throughput MAC layer multicasting in wireless networks.

